# Supporting the Integration of the Existential Dimension Into Clinical Practice: Health Care Providers' Experiences With a Meaning‐Making Synopsis of Patients Living Long‐Term With Incurable Cancer

**DOI:** 10.1002/pon.70515

**Published:** 2026-06-06

**Authors:** Anna Visser, Lenneke Post, Joost Dekker, Lia van Zuylen, Inge R. Konings

**Affiliations:** ^1^ Department of Medical Oncology Amsterdam UMC Amsterdam the Netherlands; ^2^ Cancer Center Amsterdam Cancer Treatment and Quality of Life Amsterdam the Netherlands; ^3^ Department of Spiritual Care Amsterdam UMC Amsterdam the Netherlands; ^4^ Faculty of Religion and Theology VU University Amsterdam the Netherlands; ^5^ Department of Psychiatry Amsterdam UMC Amsterdam the Netherlands; ^6^ Amsterdam Public Health, Mental Health Program Amsterdam the Netherlands

**Keywords:** existential care, existential concerns, health care providers, meaning‐making, oncology, palliative care

## Abstract

**Background:**

The existential dimension is a key aspect of palliative care but is often insufficiently integrated in clinical practice. We developed a structured meaning‐making conversation for patients living long‐term with incurable cancer led by a spiritual counsellor to support patients' meaning‐making process. To support the integration of the existential dimension, a synopsis of patient's sources of meaning and existential needs was shared afterwards with the referring health care provider (HCP) via the electronic health record.

**Aims:**

This study explored HCPs' experiences with this meaning‐making synopsis and its impact on patient care. The findings will inform further refinement of the intervention to enhance perceived benefits.

**Methods:**

Semi‐structured interviews were conducted with HCPs who referred patients for a meaning‐making conversation. A reflexive thematic analysis was performed.

**Results:**

The following themes were constructed from the interviews with HCPs (*n* = 10): providing potentially useful existential information to HCPs, supporting personalised, value‐based care through the synopsis and barriers and facilitators for using the synopsis. HCPs appreciated the synopsis for its insight into patients' sources of meaning and existential needs, for its ability to initiate conversations about meaning and its potential impact on discussing treatment options. For half of them, it helped deliver more personalised care. For some it provided reassurance that the treatment aligned with patients' wishes. The most frequently mentioned barriers to starting a conversation about meaning were lack of contact due to disrupted continuity of care and limited time.

**Conclusions:**

HCPs appreciated the meaning‐making synopsis and recognised the (potential) impact on enhancing personalised care. The synopsis can assist HCPs in integrating the existential dimension into clinical care.

## 1 Background

Existential care is widely recognised as a key component of quality palliative care, however still remains insufficiently integrated in standard clinical care [1]. Patients living with incurable cancer are confronted with future uncertainties and death, which can raise existential concerns [2, 3, 4]. Patients express the need to discuss these existential themes, such as meaning, purpose, relationships, and death [5]. These needs are not always met, as health care providers (HCPs) frequently fail to acknowledge and address existential concerns expressed by patients [6, 7].

We developed a meaning‐making conversation for patients living long‐term with incurable cancer. During this single one‐hour intervention, the spiritual counsellor explores patients' sources of meaning and priorities for the palliative phase. This can support the meaning‐making process and increase existential wellbeing [8]. It is based on a global meaning session [9, 10] to set meaningful goals for rehabilitation [10]. This format draws on the meaning‐making model of Park [11], in which global meaning is seen as the cognitive framework through which patients understand and perceive the world, and what drives their motivation. A formative, iterative research process was used to adapt this intervention for the current patient group. As part of this process, focus groups with patients living long‐term with incurable cancer, their relatives, and HCPs were conducted to assess meaning‐making needs. HCPs indicated that an overview of patients' sources of meaning could be a valuable tool, as they sometimes have no information about what matters most to a patient, or struggle to obtain this information. They found an understanding of patients' sources of meaning essential for delivering quality palliative care and informing appropriate treatment decisions.

This is in line with growing evidence that shows that the integration of the existential dimension into clinical care supports person‐centred and value‐based care [1, 12, 13]. The existential dimension encompasses, besides spirituality and religion as a way to express meaning, also value based perspectives and attitudes, and existential challenges [13]. Integration into clinical practise entails practicing compassionate, person‐centred care in which the existential domain is regarded equally important as the biopsychosocial domains. HCPs should assess patients for existential distress as well as identifying existential resources of strength. When a HCP prioritises the integration of patients' individual values, preferences, and (existential) needs into clinical decision‐making, this leads to person‐centred and value based care [14]. Interprofessional collaboration between spiritual counsellors and HCPs is an important part of this integration [14]. Differences in terminology and reluctance to share information remain barriers to such interprofessional collaboration [3, 15].

To support the incorporation of the existential dimension, we shared the outcomes of the meaning‐making conversation with HCPs, through the electronic health record (EHR). The aim of this current formative study was to explore the experience of HCPs in receiving a synopsis of patients' sources of meaning and existential needs after a meaning‐making conversation, and their perception of the impact on patient care. The findings will inform further refinement of the intervention to enhance perceived benefits [16] and support the translation into meaningful and implementable clinical practice [17].

## 2 Methods

A qualitative descriptive‐interpretative interview study with HCPs was conducted in Amsterdam University Medical Centre (UMC) and Flevo Hospital in Almere between July 2023 and May 2024 at the outpatient Medical Oncology clinics.

### 2.1 Intervention

During the intervention, patients were invited to reflect on their views of the future, including their hopes and the potential challenges they expected to face during the palliative phase. Secondly, sources of meaning were explored, concerning relationships, inner posture, world view, identity, meaningful activities and values in life [9, 10]. Finally, the patient and spiritual counsellor explored how to prioritise these sources of meaning and how they might be employed to address the previously identified challenges. More information on the meaning‐making conversation, included patients and results is published elsewhere [8].

### 2.2 Synopsis

**FIGURE 1:**
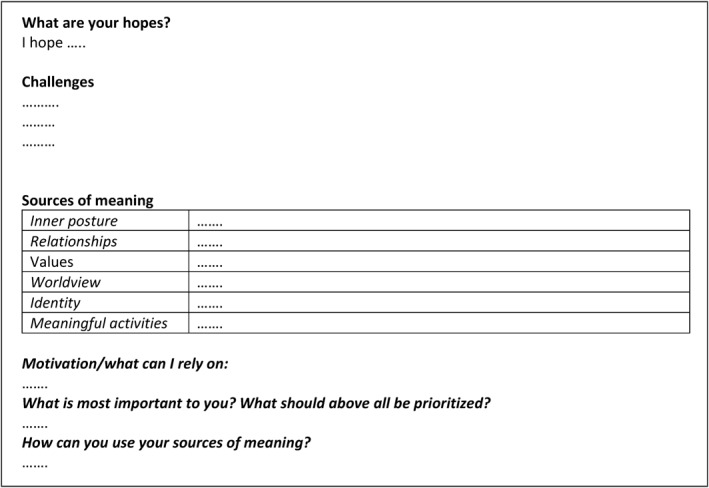
Synopsis of patients' sources of meaning and existential needs. On the dotted lines: explanation or quotation in the first person.

After a meaning‐making conversation, the spiritual counsellor wrote a synopsis of patients' sources of meaning and existential needs, the format is illustrated in Figure 1. If desired, the patient could revise the synopsis. After obtaining the patient's consent, the synopsis was uploaded to the EHR and at Amsterdam UMC a notification was sent to the referring clinician. This functionality was not available in the EHR system at Flevo Hospital.

### 2.3 Interviews

All HCPs who referred patients for a meaning‐making conversation were contacted by the researcher and invited for a semi‐structured interview and provided informed consent. HCPs were interviewed to assess their experiences with the synopsis provided, its impact on their understanding of the patients' existential needs and its impact on the care provided (see Supporting Information S1: Appendix 1). Six interviews took place in person and four through Zoom.

### 2.4 Data Analysis

Qualitative data from the interviews were audio‐recorded and transcribed verbatim. The interviews were coded by one researcher (AV) and spot‐checked by a second researcher (LP) to ensure reliability. The transcripts were subjected to reflexive thematic content analysis [18, 19]. First, the researchers familiarised themselves with the data by actively reading the transcripts (*step 1*: *familiarisation*). Coding was done both inductively (content‐driven, raised by participants) and deductively (researcher‐driven, and developed from the interview guide) (*step 2*: *initial coding*). Predetermined evaluation criteria (appreciation, benefits and implementation and application in clinical practise) were developed by the research team beforehand based on earlier research and experience and had guided the development of the interview topic guide. After coding, the researcher clustered similar codes to candidate themes to explore potential patterns of shared meaning (*step 3*: *initial theme generation*). Candidate themes were reviewed and further developed by going over all codes to see if they related to a central organising concept (*step 4*: *reviewing and developing themes*). Codes per theme and subtheme were interpreted and, if necessary, reformulated and/or merged. Themes were further refined and defined by writing theme definitions to ascertain they had a shared meaning or pattern and showed what the data of this theme entailed (*step 5*: *refining*, *defining and naming themes*). The final stage involved writing the report, where matching quotes were incorporated. This stage is used to see if the themes work and addressed the whole data set and research questions (*step 6: producing the report*).

Information power [20] was assessed as high: the study aim was narrow, focussing exclusively on HCPs' experiences receiving and utilising a synopsis; the sample was highly specific, and the interviews had a strong quality of dialogue. Consequently ten interviews were seen as sufficient for the phenomena under study and the use of reflexive thematic analysis. To enhance transparency and consistency in reporting, each theme was quantified and described using the following terms: ‘a single patient’ (one), ‘a few’ (≤ 25%), ‘some’ (≤ 50%), ‘many’ (≤ 75%), and ‘most’ (> 75%) [21].

## 3 Results

All HCPs who referred patients to the intervention (*n* = 14) were invited to participate in a semi‐structured interview. Three HCPs had relocated for their residency training and were no longer responsible for the patients they had referred. One oncologist was unavailable for scheduling an interview. The final sample consisted of ten HCPs: seven oncologists, two nurse practitioners, and one oncology fellow. Eighty percent was female, and 80 percent was affiliated with Amsterdam UMC. Each HCP had referred one to three patients.

### 3.1 Themes

**FIGURE 2:**
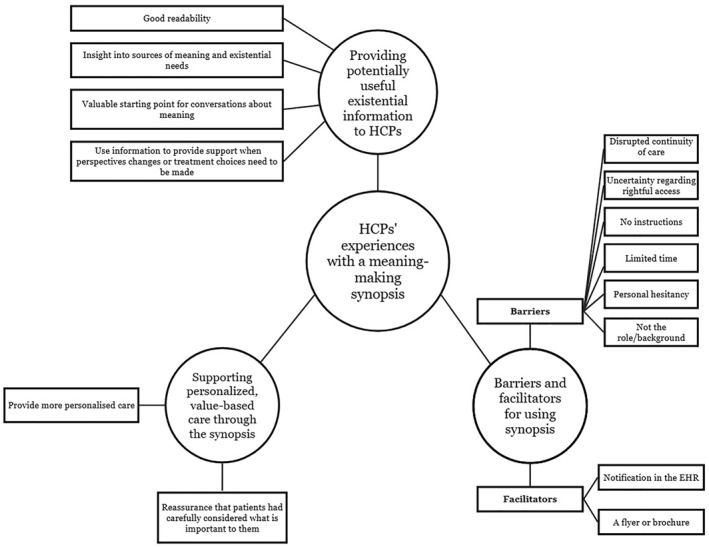
Themes and subthemes.

Figure 2 presents the identified themes and subthemes, which are further described and supported by illustrative quotations in the following paragraphs.

### 3.2 Providing Potentially Useful Existential Information to HCPs

Most HCPs appreciated the meaning‐making synopsis. It was ‘clear’, ‘concise’ and ‘interesting to read’. Many HCPs reported gaining greater insight into sources of meaning and existential needs because of the synopsis.

Many HCPs considered it to be a valuable starting point for conversations about meaning. As one medical oncologist remarked: ‘Apparently this is important for someone. I can ask about that when I don't have to talk about a scan, or when I have more time’. In addition, by providing information about quality of life, social context, and what is meaningful to the patient, the synopsis was seen as a tool to provide better support and guide more personalised and appropriate care decisions when a patient's perspective or prognosis changes.

With that one patient who really wants to maintain her quality of life, if she starts to have a lot of complaints (…) then we should discuss with her: do you still want to continue in this way? Or should we stop? I think you might want to have that conversation sooner (nurse practioner).

### 3.3 Supporting Personalised, Value‐Based Care Through the Synopsis

The information in the synopsis enabled half of HCPs to deliver more personalised care in their interactions and conversations with patients. A few knew better how to manage expectations, for example around palliative care or frequently changing HCPs. For a few HCPs the synopsis provided a deeper understanding of how to interact with their patient. As one nurse practitioner explained: ‘I think with Ms XX who indicated: I cherish hope, the glass is always half full, I think it's very important that you don't take that away from her (…) don't go into the negative, don't take away a piece of hope, while still being honest’. One nurse practitioner explained that knowing which sources of meaning supported the patient enabled her to integrate these in her care plan. ‘That piece of information about his faith came from the meaning‐making conversation. I was able to pick up on that later when I had to tell him that we wanted to stop treatment because he had so many side effects’.

Some HCPs reported that the intervention and synopsis reassured them that patients had carefully considered their preferred treatment choices, what was of value to them, and factors contributing to their quality of life. A medical oncologist noted: ‘It gave me trust that she had considered it carefully and thought about finally deciding to get treatment’. Another oncologist said the synopsis helped in accepting a patient's decision to forego treatment for a life‐threatening complication: ‘It made me feel less uneasy because I knew that she was already aware that the end would come at some point, and she had actually prepared herself for it and was at peace with it.’

### 3.4 Barriers and Facilitators of Using the Synopsis

The most frequently reported barrier to initiating a conversation about meaning after receiving the synopsis was disrupted continuity of care. This was either attributed to the fact that patients' visits were alternating between oncologists, oncology fellows, and nurse practitioners, or to HCPs transitions between departments or hospitals. Some HCPs were reluctant to discuss the synopsis due to uncertainty regarding patients' awareness of their access to this personally sensitive information. ‘I think the conversations you had were actually quite intimate in terms of privacy. I found myself thinking, Oh, is it really okay for me to read this, am I allowed to?’ (nurse practioner). A few indicated that they needed clear guidelines or instructions to act upon the synopsis.

In general, many HCPs reported that time was a limiting factor to discuss existential themes in clinical practise. A few expressed personal hesitancy because of the anticipated reaction of their patients. ‘But it can sometimes be tricky to actually bring it up, because if you do, you'll probably have to explain the whole context in great detail. Or they'll be wary of it anyway. Why are you bringing this up now?’ (medical oncologist). One oncologist did not consider it her role: ‘I realise that's not my place, because that's not my background. I care about my patients, but at the same time, I'm not the one who actually talks to them about it [meaning‐making].’

Most HCPs reported that the notification of the synopsis in the EHR was effective and prompted them to read it. Two who had not received such notifications reported overlooking synopses and expressed a preference for receiving alerts. Most HCPs suggested that a brochure could offer patients additional information and time to decide about participation. ‘Sometimes I have to discuss a lot during one consultation. It's easy if you can give people a brochure which people can read at peace at home’ (oncology fellow).

## 4 Discussion

This study explored the experiences of HCPs when receiving a synopsis of patients' sources of meaning and existential needs after a meaning‐making conversation with a spiritual counsellor. While existential care provided by spiritual counsellors in outpatient palliative care settings has been studied before, the practice of systematically sharing the outcomes of such interventions with HCPs through the EHR is novel [22].

This study suggests that sharing a meaning‐making synopsis can assist HCPs in providing more personalised care. This aligns with previous research indicating that documenting patients' preferences, values, beliefs, concerns and goals alongside medical and clinical data in EHRs is essential for enhancing personalised care and supporting clinical decision‐making in palliative care [23, 24]. Despite also recognising the synopsis's potential to facilitate conversations about existential themes and the impact on treatment decisions, many HCPs had not yet initiated them. Earlier research showed that HCPs not addressing existential themes may be due to context, culture or how HCPs view their role [25, 26]. Similarly, barriers to use the synopsis found in this study were due to either the organisation of the health care system, HCP's attitude or personal discomfort. The provision of high‐quality palliative care necessitates that HCPs feel accountable for addressing existential needs, fostering patients' sources of meaning, and incorporate these into care plans [14]. In order to provide support to HCPs in this regard, we propose several implementation strategies to enhance the utility of the synopsis.

First, ideally, a centralised location within the EHR system should be established where psychosocial and existential aspects of care can be continuously viewed and updated by all relevant HCPs. One way could be to integrate this into a standardised EHR tool for advance care planning. This approach ensures that critical information remains visible and accessible despite changes in HCPs, is not buried within extensive clinical documentation, and remains adaptable to changes in patients' existential needs over time.

Second, the inclusion of more detailed guidance in the synopsis (such as patient‐expressed wishes or interpretive notes from the spiritual counsellor) may better support HCPs in addressing existential concerns. Furthermore, it's important to ensure clear communication that patients have provided informed consent for sharing the personal information contained in the synopsis to all members of the care team. This clarification may reduce HCPs uncertainty about initiating conversations based on the information contained in the synopsis.

Third, implementing training can have a positive effect on HCPs attitudes, competencies and perceived barriers to provide attention to patients' existential needs [13, 27]. Barriers such as personal discomfort or the perception that addressing such issues falls outside the role of the physician, can also be an expression of a lack of training [26]. Furthermore, training may positively impact how HCPs perceive the time required for this integration [26]. Evidence indicates that consultations in which HCPs address patients' (existential) concerns and integrate these into the care plan do not take longer than those which do not include such concerns [28]. An interactive communication training developed in the Netherlands improved HCPs competence in recognising and addressing patients' existential concerns and incorporating them into proactive palliative care plans [29]. This may support HCPs in both identifying suitable patients for a meaning‐making conversation, addressing existential aspects of the synopsis and integrate them into care delivery.

### 4.1 Implications

As HCPs reported that the synopsis was clear, concise, and usable, this suggests that the format may contribute to reducing language‐ and terminology‐related barriers that are frequently reported by spiritual counsellors [15] and HCPs [3]. Moreover, the fact that all patients consented to sharing their synopsis suggests that concerns about breaching confidentiality may be less justified than often assumed by spiritual counsellors [15].

A larger‐scale study is needed to confirm the positive outcomes. Optimising implementation strategies will be essential to translate this approach into meaningful patient care and to establish a sustainable, valuable contribution to the integration of the existential dimension into clinical practice.

### 4.2 Strengths and Limitations

Potential selection bias should be considered as a limitation due to the format of this study. Not all eligible HCPs participated in the study, mainly due to relocated oncology fellows. Additionally, HCPs had referred patients to an existential intervention, and thus may have been more attuned to the importance of existential care compared to those who did not refer patients. This research format furthermore limits the provision of evidence of its efficacy, however this was never the study aim, which was to refine the intervention and use of the synopsis.

A notable strength is that data were collected across two distinct hospital settings, an academic hospital and a teaching general hospital, enhancing participant diversity and improving the transferability of findings to other hospital contexts in the Netherlands. The use of reflexive thematic analysis as the analytical approach is another strength, as it explicitly acknowledges that the researchers' preunderstandings inevitably shape data collection, analysis, and interpretation. Furthermore making predetermined evaluation criteria transparent enhances the overall rigour of the study. It should be noted that the primary researcher is also a clinician working at the outpatient clinic. This dual role offered advantages in terms of contextual familiarity, yet also posed the risk of assumptions during data collection and analysis. To address this, the final analytical phase was conducted collaboratively with the full research team, which included medical oncologists, a psychologist, and a spiritual counsellor.

## 5 Conclusion

HCPs appreciated the meaning‐making synopsis and recognised the (potential) impact on enhancing personalised care. The synopsis can assist HCPs in integrating the existential dimension into clinical care.

## Author Contributions

All authors contributed to the study conception and design. Material preparation and data collection was performed by Anna Visser. Data analysis was performed by Anna Visser and Lenneke Post. The first draft of the manuscript was written by Anna Visser and all authors commented on previous versions of the manuscript. All authors read and approved the final manuscript.

## Funding

This work was supported by an unrestricted grant from Gilead Sciences (Grant number: 18861).

## Ethics Statement

This study was performed in line with the principles of the Declaration of Helsinki. The Medical Research Ethics Committee of Amsterdam UMC confirmed that our study required no ethical approval.

## Conflicts of Interest

The authors declare no conflicts of interest.

## Supporting information


Supporting Information S1

## Data Availability

The datasets generated during and/or analysed during the current study are available from the corresponding author on reasonable request.
