# Supplementary material for: Supporting the Integration of the Existential Dimension Into Clinical Practice: Health Care Providers' Experiences With a Meaning‐Making Synopsis of Patients Living Long‐Term With Incurable Cancer
Source: Psychooncology. 2026 Jun 6;35(6):e70515. doi: 10.1002/pon.70515 (PMC13242079; doi:10.1002/pon.70515)
Supplement: Supplementary file 1 — Supporting Information S1 [file PON-35-e70515-s001.docx]

Appendix 1

**Topic list:**

| **Topic** | **Question** |
| --- | --- |
| 1. **Inventory** | How many patients of you had a meaning-making conversation? And do you remember who de |
| 1. **Motivation** | What was the reason to refer the patient? What did you explain about the meaning-making conversation? |
| 1. **Appreciation** | Have you read the synopsis of the conversation?  If so, what did you think of the synopsis?  If not, why not? Can you suggest any improvements that would make you read it? |
| 1. **Benefits** | Was the synopsis helpful? And how so?  Did it provide insight into the existential needs?  Did it help you to discuss meaning?  If so, how? How could it be improved?  If not, why not? What would have helped? |
| 1. **Implementation** | If we want to implement the meaning-making conversation for patients in the palliative phase in practice, what would the ideal implementation process look like? |
